# A Systematic Literature Review of the Association Between Somatic Symptom Disorder and Antisocial Personality Disorder

**DOI:** 10.7759/cureus.9318

**Published:** 2020-07-21

**Authors:** Eduardo D Espiridion, Stacie A Kerbel

**Affiliations:** 1 Psychiatry, Reading Hospital - Tower Health, West Reading, USA; 2 Psychiatry, Drexel University College of Medicine, Philadelphia, USA; 3 Psychiatry, West Virginia School of Osteopathic Medicine, Lewisburg, USA; 4 Psychiatry, West Virginia University School of Medicine, Martinsburg, USA; 5 Psychiatry, Philadelphia College of Osteopathic Medicine, Philadelphia, USA; 6 Medicine, Reading Hospital - Tower Health, West Reading, USA

**Keywords:** somatization disorder, somatoform disorders, somatic symptom disorder, antisocial personality disorder, personality disorders, serotonin, ddc gene, aadc

## Abstract

Somatic symptom disorder (SSD) and antisocial personality disorder (APSD) are found at higher rates within families compared to the general population. Both disorders are characterized by low serotonin levels, which may be attributed to polymorphisms in the dopa decarboxylase (DDC) gene. The polymorphism rs11575542 of the gene leads to decreasing the efficiency of aromatic l-amino decarboxylase (AADC) and serotonin levels in a person. The polymorphism is also associated with the development of somatic symptoms and sensation-seeking behavior, a trait underlying APSD. Hence, the role of this polymorphism as an underlying feature that may predispose a person to develop APSD or SSD should be explored further in future studies.

## Introduction and background

The relationship between personality disorders (PD) and somatic symptom disorders (SSD), previously recognized as somatization disorder (SD) or somatoform disorders by the Diagnostic and Statistical Manual of Mental Disorders, Fourth Edition (DSM-IV), has been established by the fact that those suffering from SSD often exhibit one or more PDs [1-4]. There is an increased prevalence of first-degree relatives being diagnosed with either antisocial personality disorder (APSD) or SSD compared to the general population. Both disorders are associated with low serotonin levels; therefore, genetic markers that decrease serotonin levels may play a role in the development of these disorders. The dopa decarboxylase (DDC) gene is responsible for maintaining adequate levels of serotonin in the body, while polymorphisms within the gene may decrease the level of neurotransmitters. The role of polymorphisms within the DDC and the development of APSD and SSD have not been previously explored. They may however provide an insight into the genetic link between the two disorders.

We searched Google Scholar and PubMed databases to conduct a literature review by using the keywords "somatization disorder," “somatoform disorders,” “somatic symptom disorder,” "antisocial personality disorder," "personality disorders," "serotonin", "DDC gene” "AADC." Articles found via this indexed search were examined to identify studies used in this review. This review provides an analysis of the genetic relationship between SSD and APSD.

## Review

The comorbidity between PD and SSDs has been established by multiple studies [1-4]. The increased prevalence of both SSD and APSD among family members has led researchers to believe that there is a genetic factor influencing the development of either disease [5]. Behavioral inhibition, marked by sensation-seeking, has been hypothesized to be a condition that predisposes a person to both conditions [6]. Additionally, low serotonin levels are found to be a common characteristic of both disorders. Rs11575542 is a genetic polymorphism of the DDC gene, which is found on chromosome 7; it influences the efficiency of aromatic l-amino decarboxylase (AADC), thereby decreasing a person’s serotonin level while increasing the somatization symptoms and sensation-seeking experienced by a person [7]. Sensation-seeking has been found to be positively correlated with APSD, indicating that this allele may also influence the expression of APSD [8,9]. Therefore, the polymorphism rs11575542 may indicate a genetic link between APSD and SSD that should be explored further due to the influence it has on SSD and APSD via sensation-seeking and a person’s serotonin levels.

Personality disorders and somatic symptom disorder

In those suffering from SSD, conditions such as PD including histrionic personality disorder, narcissistic personality disorder, paranoid personality disorder, borderline personality disorder, APSD, and avoidant personality disorder are found at increased rates [1-4]. Many people with SSD are likely to experience one or more of these PDs [1]. There are hypotheses regarding the comorbidity between a few of the PDs and SSD. Some people believe that somatic symptoms in those suffering from APSD are representative of malingering [3]. Additionally, those with APSD often abuse substances at higher rates, leading to increased somatization [3]. Those with an avoidant personality disorder may experience somatic symptoms due to unexpressed negative emotions, which are commonly found among those with PDs [3]. Those with a dependent personality disorder may display somatic symptoms that lead to an increase in nurture and attention from caregivers [3]. The histrionic personality disorder is proposed to be associated with an increase in somatic symptoms because the symptoms represent unconscious attention-seeking and support-seeking behaviors [3]. SSD and obsessive-compulsive personality disorder are believed to be associated since those with the PD experience rumination that may lead them to interpret minor physical changes as significant somatic symptoms [3]. The self-defeating personality disorder is associated with a deranged self-image; this prevents a person from thinking they deserve to be healthy instead of defective, leading to the development of SSD. The histrionic personality disorder is associated with SSD as hysteria is a common feature in SSD [10]. Additionally, both PD and SSD have been found to be affiliated with anxious-attachment style, which may be a reason for the comorbidity between the diseases [3]. In addition to the proposed connection between APSD, SSD, and environmental factors increasing the prevalence of the disorder, the familial link observed between those with SSD and APSD has led to the belief that there is a genetic relationship between the development of the two disorders [3]. Therefore, the genetic link between SSD and APSD will continue to be explored in the review.

Antisocial personality disorder and somatic symptom disorder

APSD and SSD have distinct presentations, leading to different diagnostic criteria as defined by the Diagnostic and Statistical Manual of Mental Disorders, fifth edition (DSM-V). The DSM-V states that for a person to be diagnosed with APSD, they must present continuous disregard for the rights of others and continually violate the rights of others, from the age of 15 years onwards [11]. Additionally, they must be at least 18 years old and must have been diagnosed with conduct disorder before they were 15 [11]. Finally, they must display antisocial behavior without the presence of behaviors related to schizophrenia and/or bipolar disorder diagnosis [11]. Within the general public, 2.1 to 3.6% meet the diagnostic criteria for APSD [12]. The DSM-V states that in order to be diagnosed with SSD, one has to have one or more somatic symptoms that disrupt daily life [13]. Those with disorders may have excessive thoughts, feelings, or behaviors that are related to somatic symptoms or other health concerns [13]. These symptoms must be present for more than six months in order to be classified as SSD [13]. Within the general population in the United States, Chinese Americans, Mexican Americans, Puerto Ricans, and Greek men are at an increased risk for SSD [3,14]. SSD has a prevalence of 0.2-3% in females and less than 0.2% in males [3]. Studies indicate that the increased prevalence of somatization seen in women is due to hypermethylation in a region in the solute carrier family 6 member 4 (SLC6A4) promoter gene [15]. The hypermethylation may alter the expression of serotonin transporter SERT, thereby increasing the prevalence of somatic symptoms [15]. While the presentations of APSD and SSD are very different, it has been reported that there is an increased prevalence of both diseases within families.

Studies have indicated an increased prevalence of SSD and APSD in first-degree relatives compared to the general population [5]. SSD and APSD are found to have a comorbidity of 3.6-63% [13]. APSD is found to be higher among children of people suffering from SSD [5]. Both diseases are found to be more prevalent in lower socioeconomic classes and often develop into chronic conditions [5].

Serotonin or 5-hydroxytryptamine (5-HT) is the neurotransmitter involved in the regulation of many behaviors and physiological processes, including mood, perception, anger, appetite, sexuality, memory, attention, and aggression [16]. 5-HT is involved in vascular function, proper cardiac function, gastrointestinal function, reproduction, and pregnancy [16]. It is released from the presynaptic nerve terminal and 5-HT transporter receives the signal [17,18]. The decreased levels of the neurotransmitter are proposed to be associated with somatic symptoms [17,18]. It has previously been found that the amount of right orbitofrontal serotonin transporter expressed in the prefrontal cortex region is positively correlated with somatization and the development of SSD [18]. Genetic factors are also found to influence somatization experienced by patients. It is believed that the genes involved in somatic symptoms are associated with pain, heart complaints, constipation, and fatigue [19]. Finally, it has been shown that selective serotonin reuptake inhibitors (SSRIs) help to reduce the symptoms associated with somatization, thereby corroborating previous studies indicating a connection between low serotonin levels and somatization [7,18].

Serotonergic pathways and 5-HT are believed to be involved in the development of APSD. 5-HTTLPR s allele and 5-HTTVNTR 12/12 genotype are polymorphisms of the SLC6A4 or 5-HT transporter gene, which are serotonin transporter genes [20]. 5-HTTVNTR is associated with impulsivity and aggression [20]. It has been demonstrated that lower levels of serotonin are associated with increased impulsivity and inhibit sensible behavior, a condition linked to APSD [21]. Deficiencies in serotonin are linked to APSD; however, a definite mechanism for the interaction between the two factors has not been previously reported [21].

The DDC gene is associated with maintaining adequate levels of monoamine neurotransmitters and the production of AADC [22,23]. There are homozygous mutations that lead to loss of function in the DDC gene, thereby depleting the pools of the decarboxylase and causing a person to develop a fatal form of infantile Parkinsonism, known as AADC deficiency [22]. The loss of function mutations have been shown to be an inherited disorder, and its symptoms include movement abnormalities, developmental delays, and autonomic system dysfunction, all of which are incompatible with normal life [7,22]. Additionally, hypotonia, oculogyric crisis, and dystonia are experienced by those with AADC enzyme deficiency [23]. When there is minimally active AADC present, the individual fails to produce adequate levels of the monoamine neurotransmitters, resulting in reduced dopamine levels and even lower serotonin levels, a characteristic of both APSD and SSD [23].

The DDC gene minor allele rs11575542 is associated with somatic symptoms [4]. This variation of the allele is the result of a missense mutation in which arginine is changed to glutamine at the 462-position located on the C-terminal of the AADC protein. The change in the amino acid does not change the level of the protein within a person. However, it lowers the activity of AADC [7]. Thus, the enzyme is saturated at lower levels, causing the amount of serotonin produced to decrease [8]. The enzyme with this mutation becomes less flexible, making it more difficult to bond to the pyridoxal phosphate (PLP) cofactor, thus reducing the ability of the enzyme to produce 5-HT and dopamine [7]. The reduced serotonin is associated with an increase in somatic symptoms [8]. The allele is codominant and affects 10% of the population [7]. The effects of this gene mutation indicate that there could be an underlying cause for the presence of somatization and disease [7].

This allele is associated with sensation-seeking, the marker which has been previously used as a measure for behavioral disinhibition and one of the original factors believed to be associated with both APSD and SSD [6]. Sensation-seeking is defined as a trait that involves people searching for varied, novel, and complex sensations and experiences, and their willingness to take social and physical risks for the sake of such experience [24]. Sensation-seeking in rats is associated with low serotonergic activity [25]. These rats were found to be more aggressive, exploratory, and socially dominant in the environments where they live [25]. Social dominance is found to be related to sensation-seeking in humans [26]. There is a statistically significant relationship between the polymorphism rs11575542 and sensation-seeking [27]. A higher degree of sensation-seeking was found in criminals diagnosed with APSD compared to the general public [8]. It has been found that sensation-seeking is correlated with APSD with an R-value of 0.21 [9]. The overlap between individuals with sensation-seeking and APSD can be seen in the fact that they both exhibit low serotonin levels and display a positive correlation.

It has been documented that there is a familial link between APSD and SSD, generating a belief that there is a common genetic factor that influences the development of either disease. Rs11575542 is a polymorphism of the DDC gene, which decreases a person’s serotonin levels. A common factor in both APSD and SSD is low levels of serotonin. The polymorphism is found in the development of SSD and sensation-seeking, a factor relating to APSD. Hence, the role of Rs11575542 as a genetic link between both APSD and SSD should be investigated further.

While we focused on the biological link between APSD and SSD, there are also psychological and social factors associated with both disease entities. Issues involving physical and sexual abuse in a child and a poor awareness of one's emotional development may be associated with both APSD and SSD [28]. In another study, evidence was presented for a genetic factor in adoptee antisocial behavior and multiple somatic complaints without medical explanations in younger female subjects [29].

## Conclusions

Based on our findings, there is an increased prevalence of a specific PD in those suffering from SSD. Additionally, both APSD and SSD have been found to have increased prevalence within families. This has led to a literature review investigating the possible genetic link within both disorders. The DDC gene polymorphism rs11575542 has been found to result in lower serotonin production, a characteristic associated with both disorders. The polymorphism has been found to be directly associated with both SSD and sensation-seeking as well as an underlying trait of APSD. Thus, this polymorphism should be investigated further to determine if there is an underlying link predisposing a person to either disease. We also recommend further studies focusing on the impact of psychological and social factors on both disorders.
